# Injectiondesign: web service of plate design with optimized stratified block randomization for modern GC/LC-MS-based sample preparation

**DOI:** 10.1186/s12859-023-05598-1

**Published:** 2023-12-20

**Authors:** Miaoshan Lu, Hengxuan Jiang, Ruimin Wang, Shaowei An, Jiawei Wang, Changbin Yu

**Affiliations:** 1 Zhejiang University, Hangzhou, Zhejiang China; 2 School of Engineering, Westlake University, 18 Shilongshan Road, Hangzhou, 310024 Zhejiang China; 3 School of Life Sciences, Westlake University, 18 Shilongshan Road, Hangzhou, 310024 Zhejiang China; 4 Institute of Advanced Technology, Westlake Institute for Advanced Study, 18 Shilongshan Road, Hangzhou, 310024 Zhejiang China; 5 Institute of Biology, Westlake Institute for Advanced Study, 18 Shilongshan Road, Hangzhou, 310024 Zhejiang China; 6 Fudan University, Shanghai, China; 7 Shandong First Medical University and Shandong Academy of Medical Sciences, Jinan, China; 8 Carbon Silicon (Hangzhou) Biotechnology Co., Ltd, Hangzhou, China

**Keywords:** InjectionDesign, Plate design, Mass spectrometry, Block randomization, Stratified balancing, Metabolomics, Web service

## Abstract

**Background:**

Plate design is a necessary and time-consuming operation for GC/LC-MS-based sample preparation. The implementation of the inter-batch balancing algorithm and the intra-batch randomization algorithm can have a significant impact on the final results. For researchers without programming skills, a stable and efficient online service for plate design is necessary.

**Results:**

Here we describe InjectionDesign, a free online plate design service focused on GC/LC-MS-based multi-omics experiment design. It offers the ability to separate the position design from the sequence design, making the output more compatible with the requirements of a modern mass spectrometer-based laboratory. In addition, it has implemented an optimized block randomization algorithm, which can be better applied to sample stratification with block randomization for an unbalanced distribution. It is easy to use, with built-in support for common instrument models and quick export to a worksheet.

**Conclusions:**

InjectionDesign is an open-source project based on Java. Researchers can get the source code for the project from Github: https://github.com/CSi-Studio/InjectionDesign. A free web service is also provided: http://www.injection.design.

## Background

Metabolomics or proteomics analysis based on mass spectrometry has great potential in scientific research. More laboratories have begun conducting metabolomics or proteomics analyses to effectively convert biological samples into digital samples. The factors that affect the final data quality, such as sample quality, experimental environment, instrument type, and sample pretreatment method, vary from laboratory to laboratory. A number of traditional approaches need to be adjusted to meet the innovation and exploration of scientific research. Still, the lack of some critical steps will undoubtedly lead to a decline in the quality of the data. Exploring and understanding the experimental steps that need to be strictly followed requires much effort but is still worth doing [1–3]. Implementing quality assurance (QA) and quality control (QC) is an essential guarantee for the reliability and consistency of data [1]. The deviation in sample distribution will cause great interference in further data analysis [2].

Randomizing the pretreated samples according to appropriate dimensions is an important method for reducing machine drift and batch effects [4–6]. A large number of studies have shown that the batch effect of samples has a significant impact on the final analysis results. [5, 7, 8]. Among the many best practice recommendations, we focus on two standard and essential algorithms: inter-batch balancing and intra-batch randomization. This two-step process can be easily manipulated by Excel or manually adjusted with simple randomization, but it becomes challenging when dealing with large queues and many participants, and a simple randomization algorithm would also introduce significant machine drift bias or batch effect bias. Operators will become prone to making mistakes under this circumstance. Different laboratories may use different models of GC/LC-MS instruments to achieve optimal results. Various kinds of specifications of plates are also involved in the pretreatment process. More and more methods or standard materials for QC are being proposed [9]. It is extremely necessary to develop the system for the inter-batch balancing algorithm and intra-batch random algorithm. which is a recommended practice that has been proposed many times [2, 10]. A stratified block randomization algorithm is an important and fast method to reduce the confounding variablesâ€™ interference [4]. Lin et al. found that 70% of the experimental works published in top journals used stratified block randomization algorithms for sample pre-processing [11].

Thousands of samples are frequently collected for a single project as the number of studies grows. This undertaking typically requires weeks or even months to complete and is unstable because it generates random sequences simultaneously. In a subsequent implementation, the distribution needs to be adjusted and reorganized. Therefore, users expect the sorting results to be traceable and modifiable. Most modern high-throughput mass spectrometers have automated sample injection processes. The position sequence of samples arranged on a plate does not correspond to the injection order of samples. The instrument is capable of independently modifying the sampling sequence, making the arrangement of samples on the plate more standardized and individualized. For instance, we can place QC samples in the same area of the left part to lessen the operational complexity resulting from QC sample interpenetration. This can significantly reduce pre-processing time and the error rate.

Here we describe InjectionDesign, a free and well-designed web service focused on the sample pretreatment stage and optimizes the plate design for the GC/LC-MS workflow. InjectionDesign mainly solves the following six problems:

1. Implementation of an inter-batch balancing algorithm and an optimized block randomization algorithm for unequally distributed samples.
2. Method for separating sample position layout from injection sequence design to better adapter to the GC/LC-MS platform.
3. Exported into a worksheet for various LC or MS instrument.
4. Interaction design based on “What You See Is What You Get” (WYSIWYG).
5. QC samples layout design and injection sequence rules.
6. Persistent storage capacity of sample injection information prepared for long-term projects.

The precision of mass spectrometry-based data analysis relies heavily on sample pretreatment strategies. Methods of randomization and balancing are rigorous and essential. However, the implementation of software for this purpose or the need for appropriate programming to obtain the proper work orders is burdensome. An efficient, free web service is crucial to solving this issue. We also provide additional downloadable local installation packages to meet the security requirements of specific laboratories.

## Implementation

### Inter-batch balancing algorithm

The inter-batch balancing algorithm is suitable for the case of a known variable. To prevent samples of the same dimension from being clustered in the same batch, which can avoid batch interference, we must evenly distribute the samples with the same 3 Dimension in each batch before injection, such as age, gender, and other factors. 81% of the laboratories reported the balancing step for samples [2].

The main detail of the inter-batch balancing algorithm lies in the treatment of the trailing samples. For example, we have a project with 50 males and 55 females. It needs to be stratified into four groups. After a simple equalization calculation, each group contains at least 12 males and 13 females. The tail sample is 2 males and 3 females. We randomized this tail of 5 samples again and interspersed them into 4 groups in turn.

### Intra-batch randomization algorithm

The block randomization algorithm combined with the stratified balancing algorithm is currently a commonly used random method in the sample pre-processing stage. Its impact on subsequent data analysis has also been specifically discussed. The need to use block randomization algorithms is necessary in most projects. The key to the block randomization algorithm is confirming the category amount of a given variable and setting the block size.

**For uniformly distributed samples**. Most biological experiments are well-designed before they begin. For example, there is a clear division between the control group and the observation group. At the same time, the number of samples in such groups is also evenly distributed, to better reduce the deviation of statistical results caused by the difference in sample distribution.

**Fig. 1:**
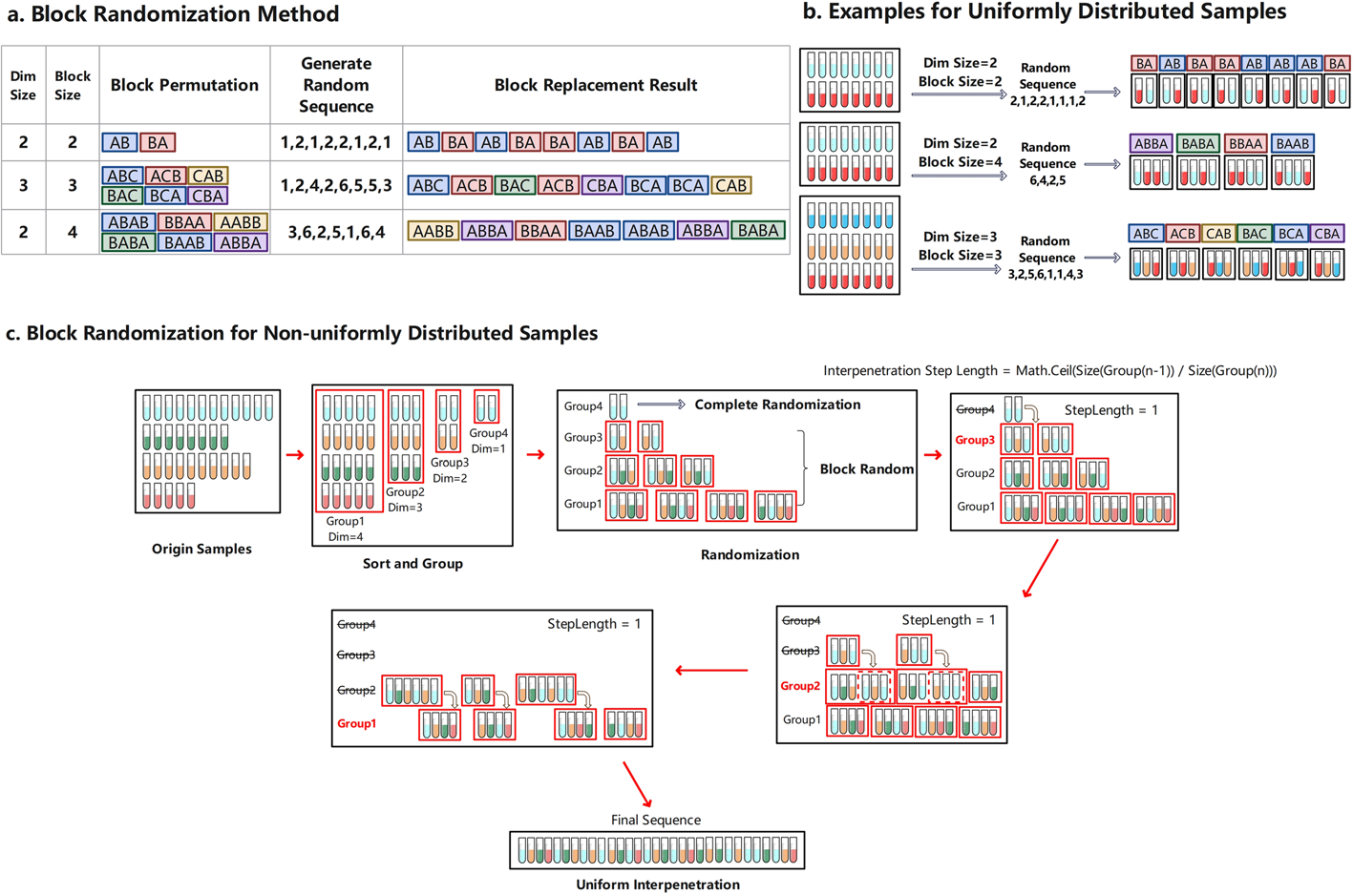
**a**The implementation principle of the block randomization algorithm. A block random factor with two variables needs to be confirmed: Dim size and block size. Then calculate the total number of block permutations according to the block random factor, calculate the number of blocks required according to the total number of samples and block size, and generate a corresponding number of random sequences. Finally, the corresponding combination is replaced in the randomized sequence to obtain the final distribution sequence. **b** The example random results under different block random factors, three factors (dim size is 2, block size is 2; dim size is 2, block size is 4; and dim size is 3, block size is 3) are shown here. **c** Optimized block randomization algorithm with group uniform interpolation method, which can maximize the uniformity of sample distribution after block randomization

When we deal with an evenly distributed sample set, we have implemented a fast block randomization algorithm. For example, if we have a project of 100 samples, 50 are in the treatment group (samples in this group are defined as A), and 50 are in the placebo group (samples in this group are defined as B). As a result, the dim size is 2. Then the block size we can choose is 2, 4, 6, .... We will use four as an example. Then the block permutation can be described as ABAB, BBAA, AABB, BABA, BAAB, and ABBA, a total of 6 combinations. Since each group contains 4 samples, a total of 25 groups are required. We need to generate a set of random number sequences in the range of 1–6. Then replace the combination of corresponding serial numbers into the sequence to obtain the final sequence. Figure 1 shows the implementation principle of the block randomization algorithm.

The block size must be set to a multiple of dim size. For example, when the dim size is 2, the block size need to be 2,4,6, etc. The larger the multiple selected, the generated result is approximately close to the completely random algorithm. Therefore, InjectionDesign uses the dim size as block size directly, which makes the final distribution most balanced.

**For non-uniformly distributed samples**. Due to the scarcity of samples, many are unevenly distributed. This is also how most projects actually stand. We are unable to apply the block-random approach directly when dealing with a queue that is not evenly distributed. This work presents an optimized block randomization approach for handling unbalanced samples. See Fig. 1c. Prior to sorting the unbalanced samples, ascending the number of samples for each categorization is necessary. Sorted samples should be vertically grouped in accordance with the maximum dimension inclusion principle. The samples in each group are dispersed equally, and we can then obtain groups with various dimensions. Except for the one-dimension group that uses the complete randomization algorithm, all the other groups use the block randomization algorithm because the sample distribution in each group is uniform. Following randomization, we then place the groups into adjacent groups. After that, by rounding down the length ratio of adjacent groups, we can determine the uniform step length for insertion. Then, in accordance with the step length, we combine the corresponding blocks of the adjacent group to create new blocks with different lengths. The cycle is thus interspersed un til the last two groups are merged.

### Intra-batch position design

The placement of the samples on the plate can be independent of the final injection sequence because the majority of contemporary mass spectrometers enable user-defined injection sequences. Delineating the sample placement region on the plate effectively can greatly improve placement accuracy and efficiency. In proteomics and metabolomics, using QC samples to minimize batch effects and enhance data quality is a popular technological method.

InjectionDesign includes common QC sample types. Solvent QC Samples, Long Term Reference (LTR) QC Samples, Blank Samples, and so on. Each laboratory has its own QC plan based on the cost of the experiments. As a result, they will have a fairly fixed and specific QC layout plan. QC samples do not enter the instrument continuously in MS-based multi-omics research. According to certain rules, QC samples are generally evenly inserted into random samples.

**Fig. 2:**
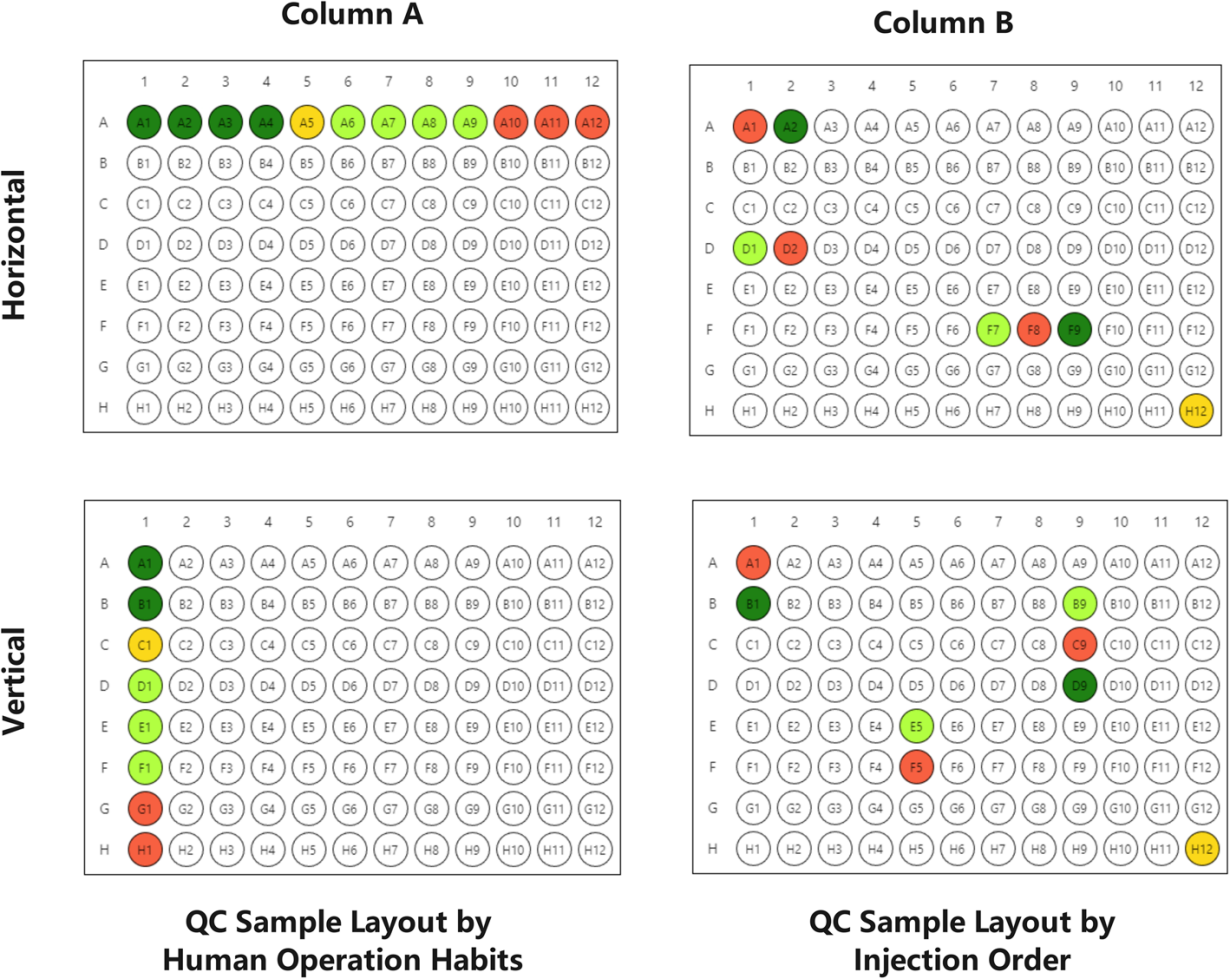
Visualization of QC samples in different layouts. It can be clearly seen that the ability to separate the plate position from the injection sequence brought by the customized injection sequence greatly improves the efficiency of sample pre-processing placement. Different colors do indicate different types of QC samples

See Fig. 2, different colors indicate different types of QC samples. By placing QC samples in a specific area of the plate, Column A employs a horizontal or vertical layout that is suitable for human operating habits. This placement method simplifies not only the placement process but also the subsequent sample traceability. Column B is placed in the injection order, regardless of whether it is horizontal or vertical, and such a QC layout will constantly be inconvenient. In laboratories without automated equipment, such a layout will result in a significant amount of extra work.

A complex process is involved in intra-batch position design. We can confirm the number of experimental samples processed in each batch after inter-batch balancing. This step must specify the location on the plate of each sample (including the QC samples) as well as the injection order. We built a workflow to make it simple to customize the steps for each laboratory. The step consists of four parts of information: Plate Specification, Plate Design Load, Predefined QC Location and Injection Order.

**Plate specification.** Users must customize the plate’s specifications. The plate information includes x- and y-axis numbers as well as x- and y-axis labels. InjectionDesign includes the following plate templates: 96-well plate template (x-axis=12, x-labels=[1,2,3,4,5,6,7,8,9,10,11,12], y-axis=8, y-labels=[a,b,c,d,e,f,g,h]) and 384-well plate template.

**Plate load capacity.** Users should determine the number of QC samples of each type used. Select the custom plate’s maximum experimental sample capacity as well. We have built in many of the QC sample types suggested by mQACC [9], as well as custom sample types to allow for possible extensions for special samples in the lab.

**Predefined QC location.** The predefined injection location is mainly for QC samples. QC samples are usually placed in the first or last column of the plate. Because there is no absolute relationship between the injection location and the injection order, it can be set according to the operatorâ€™s habits to reduce the difficulty of sample transfer between plates.

**Pre-interpolation of QC.** The QC pre-interpolation configuration is performed after the randomization of the samples. QC samples are usually interpolated evenly among the experimental samples. It will randomly arrange all experimental samples before inserting QC samples in a circular fashion to form a new injection sequence.

### Technology framework

InjectionDesign is constructed using a pattern that separates the front and back ends. The front end is written using the React-based AntDesign framework. The backend is built using Java-based SpringBoot, and the database is built using MongoDB [12]. The diagram presentation framework used is echarts [13]. All the technologies used are free or open-source products. Teams with strict data security requirements can also deploy InjectionDesign in a private cloud environment.

## Results

### Main workflow

**Fig. 3:**
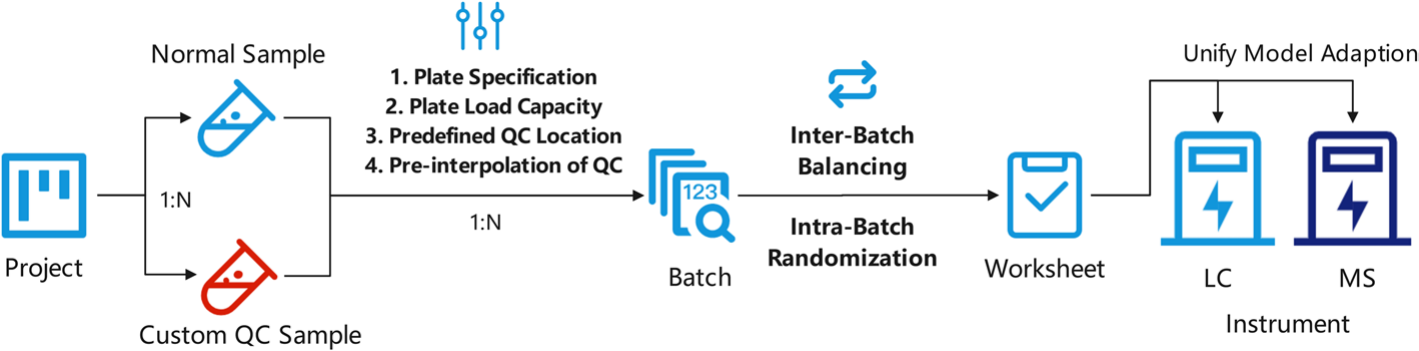
The main flow of InjectionDesign, which contains the implementation order of all the methods mentioned in the section

A special workflow is formed as Fig. 3. The user first needs to create a project to store all the imported sample information. Alternatively, samples can be imported directly via Excel. The system will automatically create the associated project. InjectionDesign provides the ability to store samples persistently. As a result, uploaded samples can be accessed and processed repeatedly, even after worksheets are generated.

### Comparison of similar tools

At present, there are already some tools for plate design such as OSAT [14], Well Plate Maker(WPM) [15], Omixer [16], PlateDesigner [17], PlateEditor [18] and PLAID [19]. Although the plate design takes up little time in the whole experimental process, the layout design and the injection sequence of the experiment have a significant impact on the final identification. Therefore, unless the Standard Operating Procedure(SOP) is so complex that it is necessary to customize a plate design system. Most researchers would like to have a system that they can use quickly and easily. Web services are one of the most suitable technology for such tools. In the above tool, PlateDesigner, PlateEditor and PLAID provides web-based visualization interface and WPM provides a local visualization interface to make plate design easier. OSAT and Omixer are developed in the R language, and they focus more on building algorithms that eliminate batch effects between samples. These two tools are still an excellent option for researchers with experience in R language development. OSAT is a tool for designing genomic injections that takes consideration of effective intra- and inter-batch randomization strategies. Omixer deals with batch effects by shuffling the sample 1000 times and picking the best arrangement based on the one with the smallest sum of correlations between biological and technical factors. In addition to PlateEditor, the other five tools put a lot of effort into solving the batch effect problem. PlateDesigner allows users to balance and randomize the distribution of samples among plates based on a specific dimension. The randomization algorithm is however not described in detail. PlateDesigner only supports the definition for the number of quality control(QC) samples, but not for the QC type. Besides the basic plate design functions, PlateEditor also provides many detailed functions, such as concentration curve configuration, specific sample configuration, multiple plate support, statistical analysis, and other functions. The interface of PlateEditor is very rich and easy to use. Unfortunately, it doesn’t support sample randomization. WPM does not provide web services and requires R language basics to build and start properly. However, it provides a method of setting the constraint hole location, which can replace the setting function of the QC samples. The randomization method is also an arrangement algorithm based on location constraints, not a block randomization algorithm. PLAID directly finds the optimal solution for multiple rules by introducing the solver algorithm. Compared with Omixer’s method of randomizing 1000 times to find the optimal solution, PLAID has higher stability and accuracy.

In the experimental design based on GC/LC-MS, the modern mass spectrometer can arbitrarily control the sample injection sequence rather than the well plate position. Therefore, the sample layout and injection sequence can be designed separately. This function is commonly provided by the mass spectrometer, and it is also the reason why InjectionDesign is more suitable for the mass spectrometer. We use Separation of Sequence & Layout in the table to emphasize that.

In contrast, laboratories with GC/LC-MS platforms usually have several kinds of GC/LC-MS instruments. Different instruments have strict requirements for the head of the imported table. If the system does not support customized headers for different GC/LC-MS instruments, then each export file requires the researcher to manually adjust the headers. This leads to lower efficiency. Therefore, it is beneficial to offer the ability to export worksheets for numerous GC/LC-MS instruments. We use Customization Worksheet in the table to emphasize that.

During the sample pre-processing in proteomics and metabolomics, different kinds of quality control (QC) samples must be clearly defined and systematically interspersed throughout the sampling queue. QC sample Support means that the system allows customization of QC samples, including the QC sample type, sample number, and fixed position of QC samples in the plate. InjectionDesign provides a randomization algorithm to solve batch effects and rich plate design features. It also adds proprietary optimization features for GC/LC-MS instruments.

**Table 1:** The features of similar tools in the sample injection process for GC/LC-MS platform

Features	WPM	Plate editor	Plate designer	PLAID	Omixer	OSAT	Injection design
Web service	x	$\checkmark$	$\checkmark$	$\checkmark$	x	x	$\checkmark$
GUI	$\checkmark$	$\checkmark$	$\checkmark$	$\checkmark$	x	x	$\checkmark$
Resolving batch effect	$\checkmark$	x	$\checkmark$	$\checkmark$	$\checkmark$	$\checkmark$	$\checkmark$
QC samples support	$\checkmark$	$\checkmark$	$\checkmark$	x	$\checkmark$	$\checkmark$	$\checkmark$
Separation of sequence and layout	x	x	x	x	x	x	$\checkmark$
Customization worksheet	x	x	x	x	x	x	$\checkmark$

See Table 1. We compared InjectionDesign to several other visualization tools. InjectionDesign develops the interface by the way of What You See Is What You Get(WYSIWYG). It displays the effect of random result in real time and has a well-design visualization.

Most tools handle batch effects as a core function in general. All tools provide the following features: Worksheet export; Multi-format plates support; Available user manual; Free to use; On this basis, additional functions such as the layout design of concentration curve samples and the uniform layout of plates is provided to improve the ease of use of the tool. InjectionDesign adds an optimized solution for GC/LC-MS platform plate design, hoping to provide a free tool with a better operating experience.

### Case study

We created a list of 1300 samples, each of which included the following information: gender (615 Male, 434 Female, 251 Unknown), whether treatment was used (650 treatment, 650 placebo), and age group (169 Kids, 351 Teenager, 481 Middle-Aged, 299 Older).

**Fig. 4:**
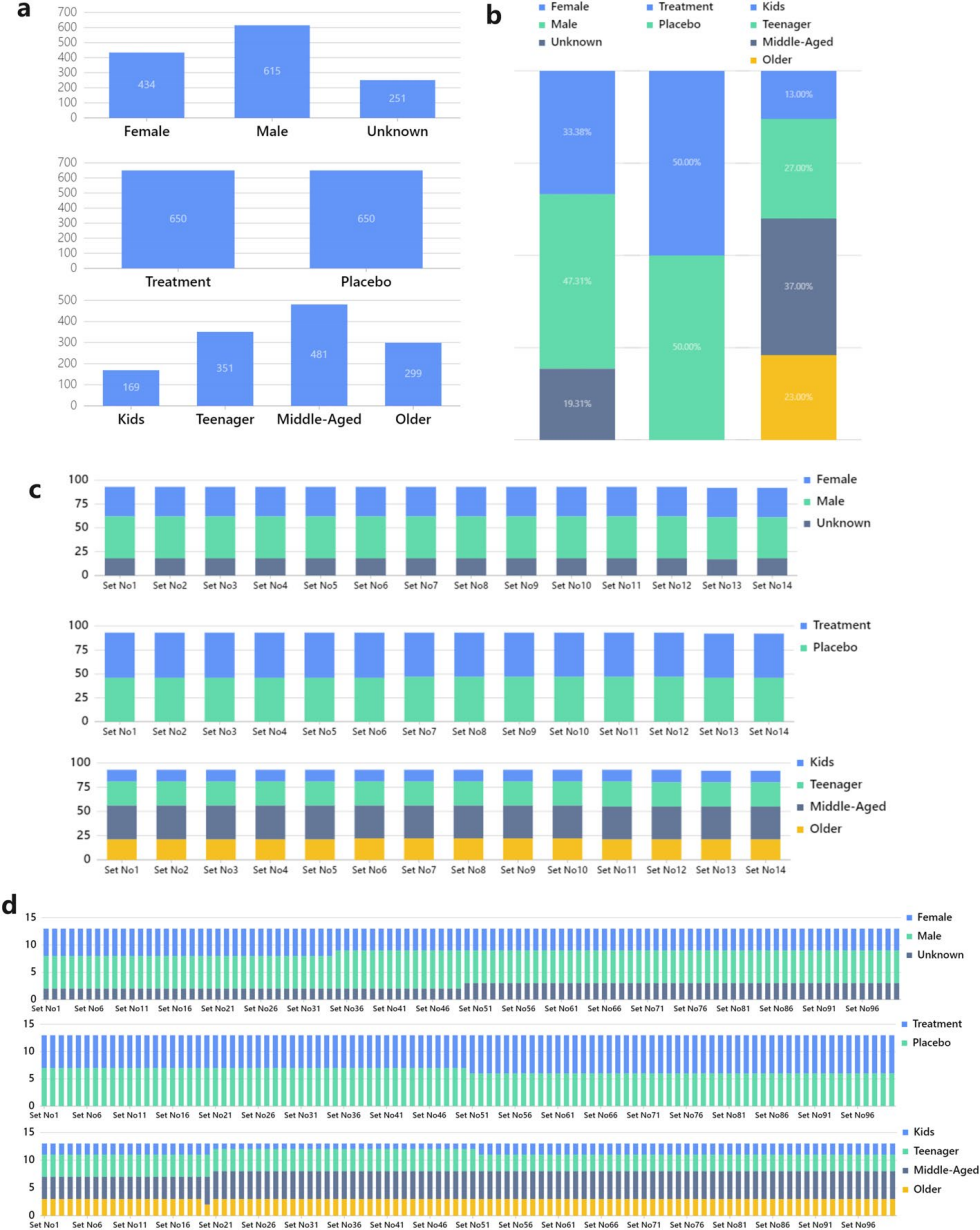
**a** Distribution of 1300 samples in three different dimensions. **b** Sample distribution in a stack bar viewer in three distinct dimensions. **c** and **d** show the proportion of samples distributed on each 96-well plate after using the inter-plate balancing algorithm. **c** specify the maximum number of samples on plate as 96. **d** specify the maximum number of samples on plate as 13. It is clear that, whether the distribution is balanced, the inter-plate balancing procedure works well for equating the sample partition

see Fig. 4a, b. They represent two-categories, three-categories and four-categories, respectively. The two categories are uniformly distributed. We chose a 96-well plate as the processing plate. The maximum number of samples that can be placed on each plate was also initially set to 96.

#### Inter-batch balancing result

Due to the sufficient sample size of individual plates, as seen in Fig. 4c, the distribution of a single plate is extremely close to the overall ratio. To test the effectiveness of the balancing algorithm with a small number of samples, We set the maximum capacity of the plate to 13 (just for convenience of division, setting the number to about 10 can get a similar result). see Fig. 4d. Although the very small number of individual plates leads to a significant imbalance in the proportion of unevenly distributed samples, the distribution of samples varies very little across the majority of the plate, but some plates do have slightly larger deviations. Naturally, we do not advise using a stratification sample size that is too small.

Additionally, InjectionDesign offers the complete randomization algorithm, which does not stratify by dimensions but is completely random. This method works well for unidentified interferences or when simulating the sample’s actual distribution on a small scale.

#### Intra-batch randomization result

The block randomization algorithm’s greatest benefit is that it guarantees that, in the event of a balanced distribution of samples, there will be absolutely no aggregated distribution. The treatment/placebo dimension was balanced, and since the block size is 2, theoretically there were no three consecutive samples of the same group in the results.

**Fig. 5:**
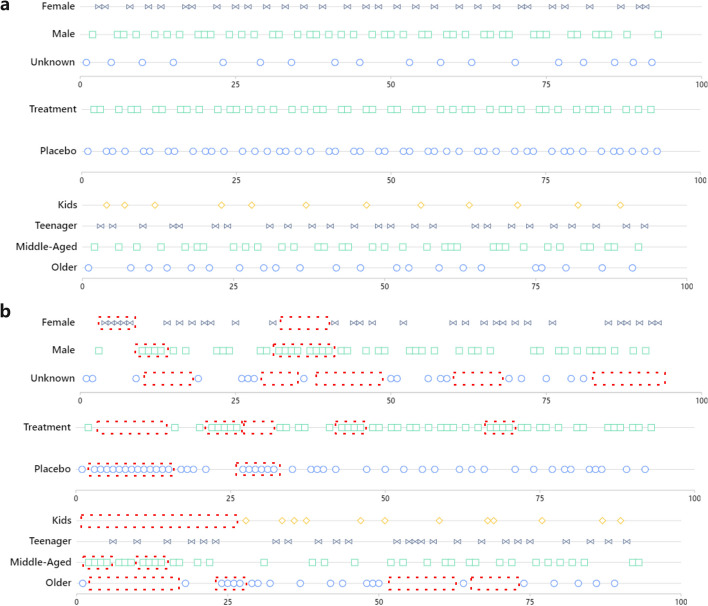
**a** Results of block randomization in three dimensions, the overall distribution of the data showed a clear balance. **b** Complete randomization result with three different dimensions. The area delineated by the red dashed box shows a clear random clustering of the sample distribution

see Fig. 5a, In the final results, all samples were evenly distributed, and there were no three consecutive samples in the same group. In the remaining unbalanced dimensions, the optimized block randomization algorithm also achieves good results. All samples are relatively uniformly distributed but still exhibit complete randomness in a small range.

Like the inter-batch balancing algorithm, InjectionDesign also provides a completely randomized algorithm for intra-batch samples. The complete randomization algorithm generates a large number of aggregated features with high probability. see Fig. 5b, the red box is the part with the more obvious aggregated distribution. The optimized block randomization algorithm has significant distribution control.

### Main steps

#### Upload samples

**Fig. 6:**
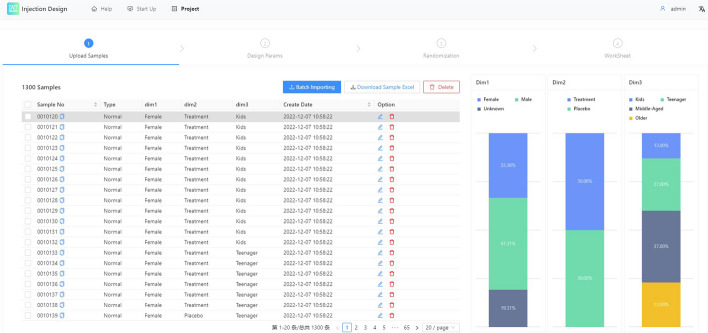
Step of sample uplaoding and sample distribution visulization

See Fig. 6. The use of InjectionDesign is very simple. First, obtain the Excel template from the web. The sample ID, sample type, three covariates, and other relevant data are all included in the template. Following a successful upload of the sample, InjectionDesign will provide the distribution of each and every variable. After upload, users are permitted to edit the sample data.

#### Design parameters

**Fig. 7:**
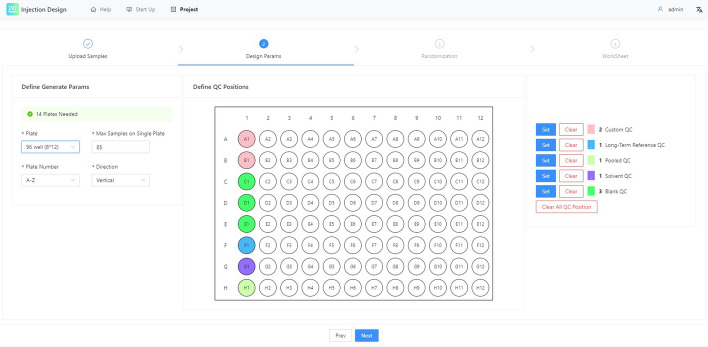
Step of setting the parameters of the project. Set parameters for the plate layout, QC types, and QC samples, which is a WYSIWYG interface

See Fig. 7. Setting the total number of samples in a single batch, the location of internal standard samples, the placement direction, and other details are the major tasks for this stage. due to the possibility of an uneven injection plate. On various plates, the horizontal and vertical coordinates could be represented by numbers or letters. The user is permitted to change the coordinate system of each plate, ensuring that the final worksheet can be accurately recognized by LC or MS instruments.

#### Randomization

In the randomization step, users can adjust the parameters of the stratified algorithm, the randomization algorithm, and the stratified and randomization dimensions.

**Fig. 8:**
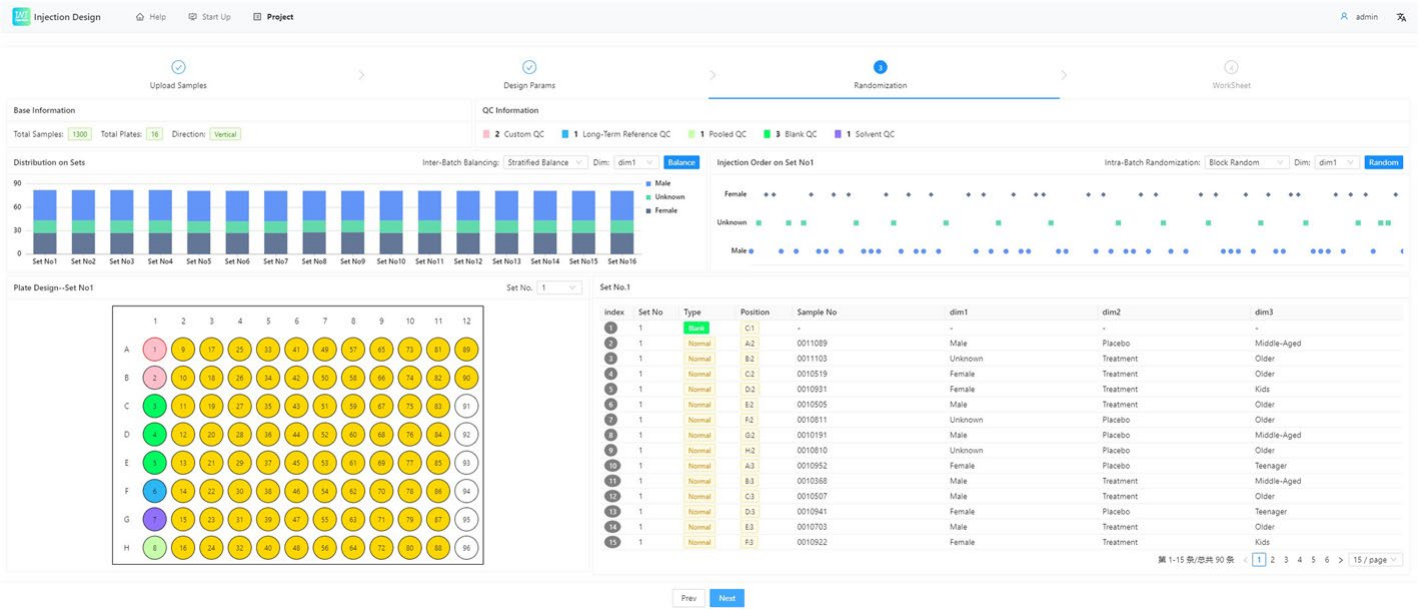
Step of inter-bacth balancing and intra-batch randomization. The randomization step is a WYSIWYG interface. Whenever the algorithm is switched or refreshed, the final injection sequence and dimension distribution map are displayed in real time

See Fig. 8. The results of the randomized distribution are immediately displayed in a visual graph after the parameters are specified, considerably helping the user understand the final result of the randomization.

#### Worksheet overview

**Fig. 9:**
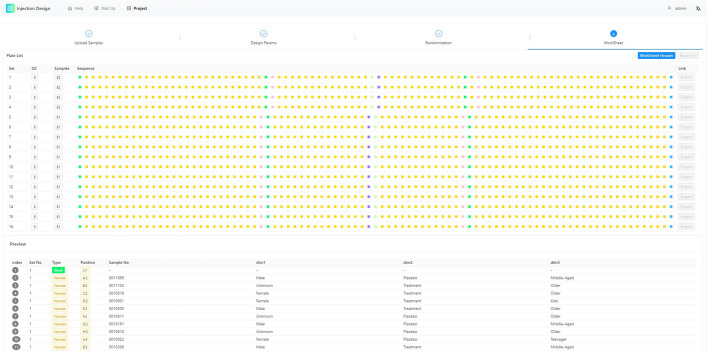
Step of the overview of randomization result. This interface mainly shows the distribution of QC samples in the injection sequence

See Fig. 9. According to the automatic algorithm, the internal standard sample will be evenly interleaved with the experimental sample. Users can see the visual effect of the final sample injection order at this phase.

#### Export for GC/LC-MS

The worksheet fields of different LC or MS instruments are usually different. Users can export it directly and modify it in Excel. InjectionDesign also provides the function of a field mapper.

**Fig. 10:**
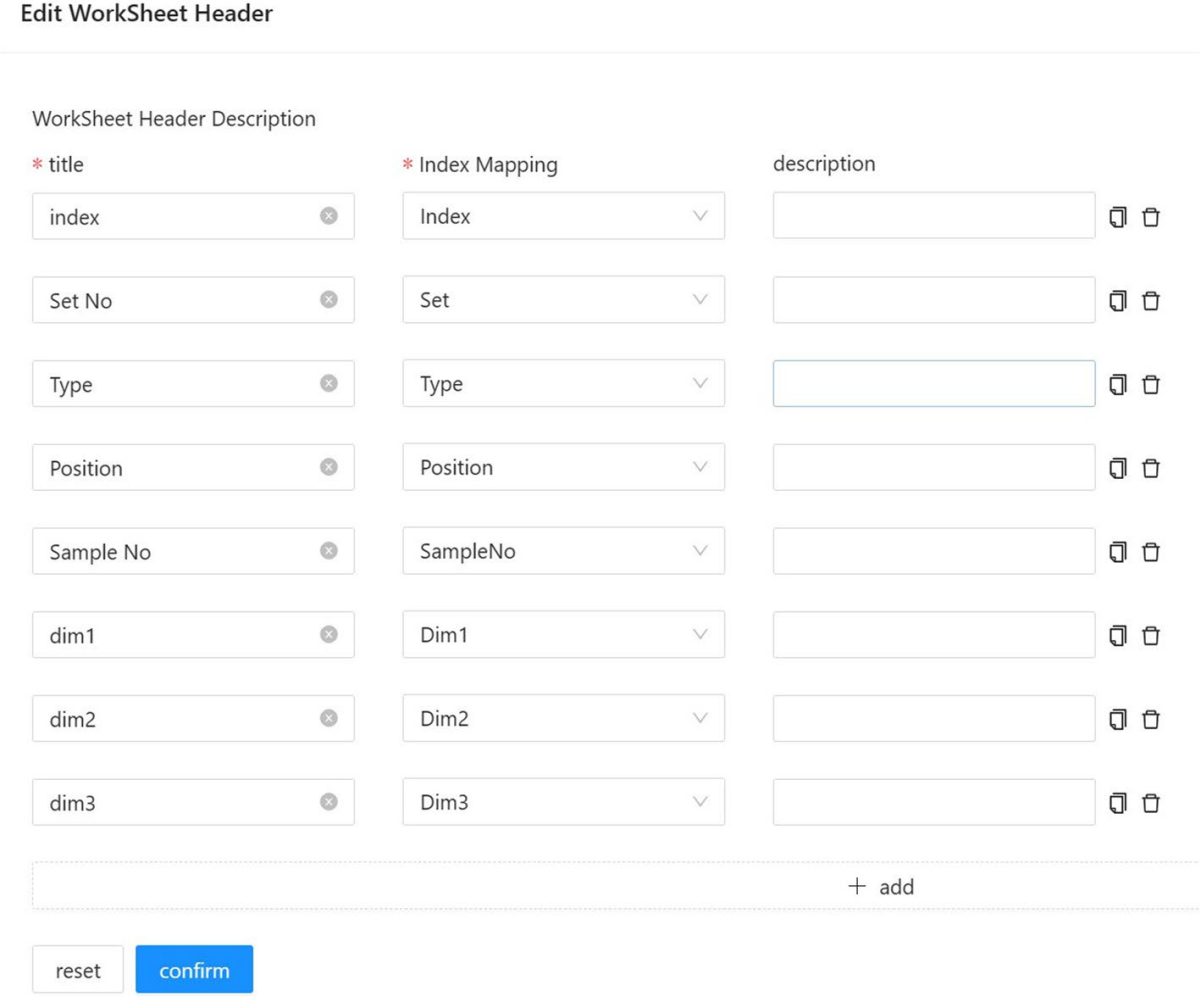
Step of the export for GC/LC-MS worksheet. Worksheet formats differ between LC or MS instruments. A meter head adapter is offered by InjectionDesign to export worksheet for various instruments

See Fig. 10. The worksheet can be exported after the configuration mapping field is set correctly. This allows exported worksheet to be imported directly into these instruments, reducing errors that can be introduced by manual operations.

## Conclusions

The sample processing flux in the laboratory is increasing as metabolomics and proteomics technology evolve. Some laboratories will install automated pre-processing equipment, but this is frequently costly. InjectionDesign focuses on the quality control steps of sample pretreatment, which can significantly reduce non-standard operations caused by manual processes. Despite the fact that many detailed steps are carried out in the form of SOP. Too many manual interventions, such as sample transfer between plates or sample injection tube label identification, will still result in errors. This will also be the source of power for InjectionDesign’s iterative process.

The stratified block randomization algorithm is suitable for most sample pre-processing. However, the block randomization algorithm completely averages out the distribution of the sample features, thus losing some of the numerical characteristics that are naturally present in the sample distribution. Although the complete randomization algorithm provided by InjectionDesign can be used to some extent for such scenarios, but it is not a perfect solution.

The optimized block randomization algorithm can better balance the distribution of non-uniform samples. However, when there are too many groups in one dimension, the averaging step calculation method may cause some local aggregation problems under some special numerical distributions.

There are many established methods and procedures in current MS-based pre-treatment experiments. However, new methods and procedures are being developed all the time. For example, new QC samples and new injection rules may emerge. InjectionDesign also hopes to gain access to more of the lab’s innovative methodologies after the release, and to update them in the web service.

Sample pretreatment involves operators, samples, consumables, and instruments, the four most essential elements. This makes the operation complicated and prone to human errors. InjectionDesign provides various laboratories with rich extensibility by customizing QC types, randomization methods, plate types, export fields, and injection rules. To enable each laboratory to try InjectionDesign quickly, we provide a free web service with suitable visual interfaces.

Because of the data security requirements in some laboratories, InjectionDesign also provides the ability to install locally. A complete installation package is provided on Github. InjectionDesign relies on MongoDB and OpenJDK and is a cross-platform deployable application. Users can also make suggestions directly on GitHub, and the development team will respond quickly.

## Data Availability

Project name: InjectionDesign; Project home page: GitHub home page: https://github.com/CSi-Studio/InjectionDesign; Online service: http://www.injection.design/; Operating system(s): Windows, Linux, MacOS; Programming language: Java, TypeScript; Other requirements: None; License: MulanPSL-2.0 license; Any restrictions to use by non-academics: None. Data available in Case Study section: The data utilized in this section is generated solely through computer simulations and does not involve real-world data. It can be downloaded from https://github.com/CSi-Studio/InjectionDesign/blob/main/SampleTemplate.csv.

## Abbreviations

LC: Liquid chromatograph
QC: Quality control
QA: Quality assurance
MS: Mass spectrometry
mQACC: Metabolomics Quality Assurance and Quality Control Consortium
LTR: Long-term reference
WYSIWYG: What You See Is What You Get
SOP: Standard operating procedure
WPM: Well plate maker
